# Ammonia vs. Lactic Acid in Predicting Positivity of Microbial Culture in Sepsis: The ALPS Pilot Study

**DOI:** 10.3390/jcm7080182

**Published:** 2018-07-26

**Authors:** Yazan Numan, Yasir Jawaid, Hisham Hirzallah, Damir Kusmic, Mohammad Megri, Obadah Aqtash, Ahmed Amro, Haitem Mezughi, Emmon Maher, Yonas Raru, Jamil Numan, Sutoidem Akpanudo, Zeid Khitan, Yousef Shweihat

**Affiliations:** 1 Internal Medicine Department, Marshall University, Huntington, WV 25755, USA; numan@marshall.edu (Y.N.); Jawaidy@marshall.edu (Y.J.); Hirzallah@marshall.edu (H.H.); Kusmic@marshall.edu (D.K.); Megri@marshall.edu (M.M.); Aqtasho@marshall.edu (O.A.); Amro@marshall.edu (A.A.); Mezughi@marshall.edu (H.M.); Maher11@marshall.edu (E.M.); Raru@marshall.edu (Y.R.); numanj@marshall.edu (J.N.); Akpanudo@marshall.edu (S.A.); 2 Nephrology Department, Marshall University, Huntington, WV 25755, USA; zKhitan@marshall.edu; 3 Pulmonary & Critical Care Department, Marshall University, Huntington, WV 25701, USA

**Keywords:** ammonia, sepsis, microbial cultures, SIRS, bacterial infections, lactic acid

## Abstract

Objective: The use of serum ammonia as a novel marker for sepsis compared to lactic acid levels in intensive care unit (ICU) patients. Design and Interventions: Single arm, prospective clinical trial to collect arterial blood samples from patients with sepsis. Serial ammonia and lactic acid levels were sent every six hours for a total of three days. Measurements and results: Compare mean levels of ammonia and lactic acid in terms of diagnosing sepsis and patient outcome, including length of stay and mortality. A total of 30 patients were enrolled in the pilot study. On admission, mean ammonia level was 35.7 μmol/L and lactic acid was 3.06 mmole/L. Ammonia levels checked at the end of day 2 (ammonia 2-4) and the beginning of day 3 (ammonia 3-1) were higher in patients who had a microbial culture-proven sepsis (*p*-values 0.029 and 0.002, respectively) compared to those without culture-positive sepsis. Ammonia levels did predict a longer hospital stay; ammonia level of more than 40 μmol/L had a mean hospital stay of 17.6 days vs. patients with normal levels who had a mean hospital stay of 9.62 days (*p*-value 0.0082). Conclusion: Elevated ammonia level can be a novel biomarker for sepsis, comparable to conventional markers. Ammonia levels have a prognostic utility as elevated levels were associated with longer hospital stay.

## 1. Introduction

The Surviving Sepsis Campaign guidelines (SSG) for management of sepsis and septic shock defined sepsis in 2016 as a life-threatening organ dysfunction caused by a dysregulated host response to infection. Septic shock is a subset of sepsis with circulatory and cellular/metabolic dysfunction with a high risk of mortality [1]. The previous definition of sepsis was systemic inflammatory response syndrome (SIRS) with an identifiable source of infection. At the cellular level, sepsis is a heterogeneous disease process causing deranged oxygen metabolism. Sepsis is a distributive shock secondary to microcirculatory heterogeneity, leading to a delivery and consumption mismatch at the cellular levels secondary to ischemic and cytopathic hypoxia [2].

In 2016, SSG incorporated serum lactic acid as a marker of systemic hypoperfusion [1,3]. However, in recent literature, lactic acid has been scrutinized because of non-anaerobic causes of hyperlactemia in sepsis [3]. In this study, we aim to determine the significance of elevated serum ammonia levels during hospitalization in sepsis and its effects on patient outcomes. We also evaluate the sensitivity and specificity of ammonia levels in predicting true sepsis as defined above.

Our rationale for studying ammonia primarily stems from the proximity of the urea and Krebs cycles in mitochondria. We have examined both cycles and hypothesized that: (1) sepsis affects the nitrosylation of amino acid residues of the electron transport chain, and perivenous glutamine synthase leads to the hyperammonemia; (2) urease-splitting bacteria are associated with hyperammonemia in noncirrhotic patients [4].

## 2. Methods

The ALPS (Ammonia vs. Lactic acid in Predicting Sepsis) pilot study is an investigator-initiated, single arm trial. The sample size was estimated to be 30 patients for a pilot study. The study was carried out according to the Code of Ethics of the World Medical Association (Declaration of Helsinki), and informed consent was obtained. An order set was created in the electronic medical record for timed ammonia and lactic acid collections. Ammonia and lactic acid levels were designated based on the day collected and the numbered collection for that day, for example, 1-2 meant the second level drawn on day 1. The patients were recruited from the emergency department of a Marshall University-affiliated hospital. We selected potential enrollees as those who were started on a sepsis order set/protocol in their charts (which entails collection of microbial cultures and starting broad spectrum antibiotics) on the basis of a presumed diagnosis of sepsis by the emergency physicians. Once admitted to the hospital, the patients were evaluated by internal medicine residents who confirmed eligibility and placed the ammonia/lactic acid study order sets. Once the patients were enrolled, the phlebotomist collected a blood sample every six hours for three days. To ensure accurate test levels, all samples were transferred on ice to the hospital laboratory. Lactic acid levels (mmole/L) and ammonia levels (μmol/L) were tested simultaneously on the same blood sample. We considered a normal lactic acid level as less than 1.2 mmole/L and a normal ammonia level as less than 40 μmol/L. Basic laboratory testing, including white blood cell counts, procalcitonin levels, and a source of infection, were collected from the charts. The Acute Physiology and Chronic Health Evaluation II (APACHE II) scores were calculated to estimate mortality during hospitalization. An unpaired *t*-test was used to test different mean levels of ammonia and lactic acid.

Patients were divided into two groups in accordance with the Surviving Sepsis guidelines. Patients who had SIRS and positive cultures (confirmed sepsis group) were compared to those with SIRS and negative cultures (non-confirmed group). *Staphylococcus epidermidis* microbial cultures were excluded and considered as contaminant. The treating physician chose the broad-spectrum antibiotic regimen. A subset of infections was considered atypical if they were positive for an extended spectrum beta-lactamase (ESBL) producing organism or fungal infection.

## 3. Results

A total of 30 patients had a suspected diagnosis of sepsis on admission and were enrolled in the study. The mean age of the patients was 58.3 years. A majority (63.3%) of patients were male. A total of six patients had diagnosis of chronic liver disease and suspected sepsis. Mean ammonia level on admission was 35.7 μmol/L, and mean lactic acid level on admission was 3.06 mmole/L. Mean white blood cell count on admission was 17.400 × 10^3^ cells μL. The mean APACHE II score was 20, with a corresponding mortality of 33%. Mean procalcitonin level on admission was 7.2 ng/dL. A total of nine patients (30%) died during the study (all deaths were after two days of enrollment), and the remainder survived to discharge. Table 1 outlines patient characteristics grouped by ammonia level of more than 40 μmol/L. Table 2 outlines the sources of infection in patients enrolled in the study. No difference in the mean ammonia levels between survivors and non-survivors was found. Nineteen patients (63.3%) had a positive microbial culture and were regarded as confirmed sepsis. The remaining 11 patients with a negative microbial culture were ruled out for having sepsis and received an alternate diagnosis. The following organisms were isolated from those with a positive microbial culture: 13 patients had gram-negative bacterial infection (seven *klebsiella organisms*, three *Escherichia coli*, and three *Serratia marcescens*); the rest had streptococcus pneumonia (three patients) and candida infection (two patients). Two patients had more than one isolated organism. Out of the 19 patients, five patients (16.7%) grew ESBL and candida from their cultures, and their antibiotic regimen was changed appropriately to cover these organisms, i.e., meropenem and caspofungin.

**Figure 1:**
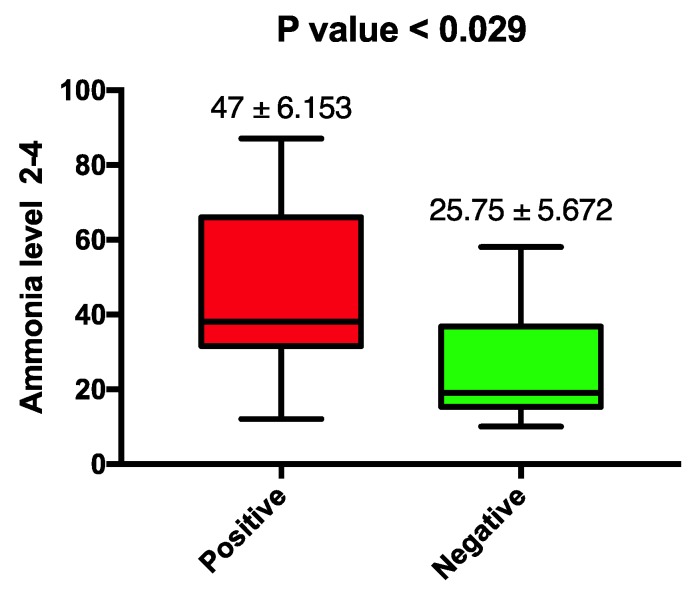
Microbial culture and mean ammonia level 2-4. Microbial cultures include blood, urine, respiratory secretions. Ammonia level 2-4: ammonia level checked at the end of day 2 of enrollment or admission to hospital. Mean ammonia level is reported in μmol/L. Values are presented as the following: mean ± standard error of mean (SEM). Statistically significant value is *p* < 0.05.

**Figure 2:**
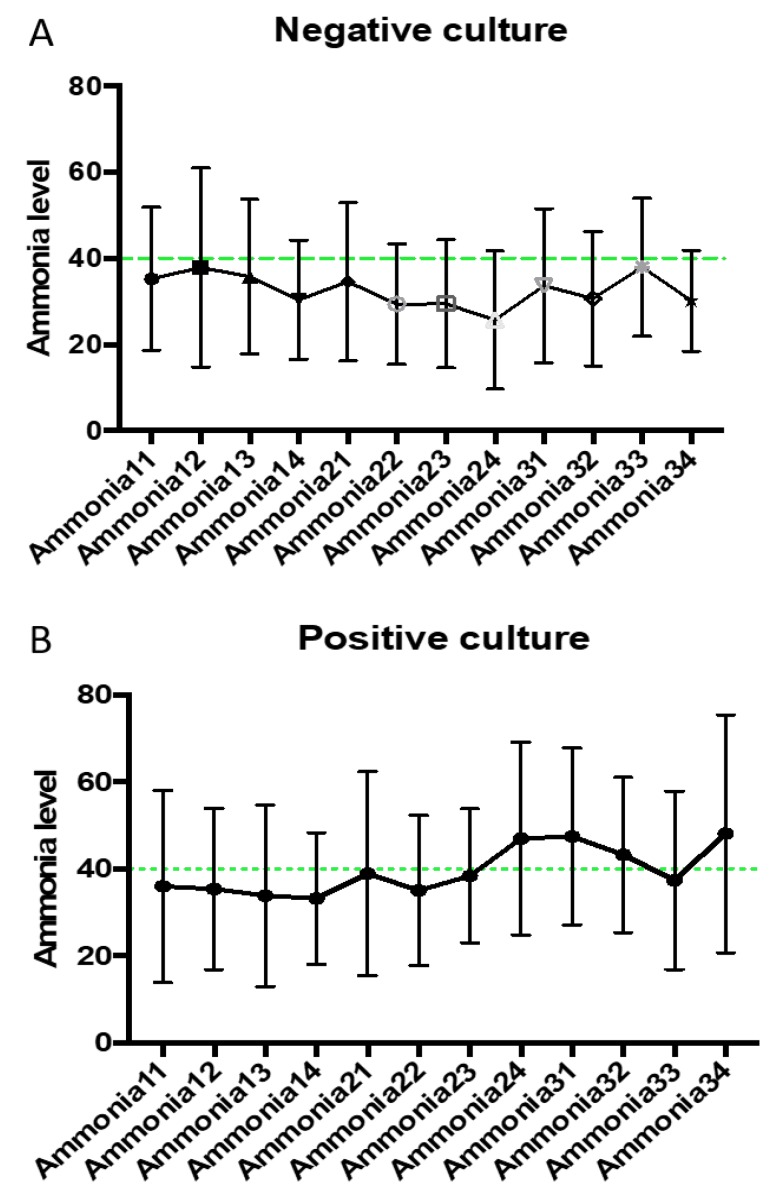
Average ammonia level for all patients with (**A**) negative and (**B**) positive microbial culture, respectively. Mean value at each set point of day for all patient is represented in mathematical closed shape (e.g., filled closed circle is representative of ammonia level 1-1). Green dashed line represents normal ammonia value. Vertical lines represent standard deviation for each value. Mean ammonia level is reported in μmol/L.

Ammonia levels checked at the end of day 2 (ammonia level 2-4) were higher in patients who had a diagnosis of sepsis (47 ± SEM 6.1 vs. 25.75 ± SEM 5.6). Furthermore, the first level check at day 3 (ammonia level 3-1) was consistent with ammonia level 2-4 (47.5 ± SEM 1.69 vs. 33.7 ± SEM 2.5). This finding was statistically significant, with *p*-values 0.029 and 0.002, respectively, as illustrated in Figure 1. This was further supported with the upward trend of serial ammonia levels checked for patients with sepsis compared to those in whom the diagnosis of sepsis was ruled out and antibiotic treatment was stopped. Figure 2 are a graphical demonstration that highlight the marked differentiation between ammonia curves in the two patient groups. We noticed a particular elevation in ammonia levels 2-4 and 3-1 in patients with positive microbial cultures. This is in concordance with the statistical significance detected above. The vertical bars represent the standard deviation for each ammonia value, and no statistical significance was found. The remaining ammonia levels were not significantly different between the two patient groups.

The mean ammonia level for alive patients was 40.1 μmol/L vs. 35.2 μmol/L for patients who died during the study; therefore, increased ammonia levels did not predict mortality. However, ammonia levels did correlate with longer hospital stay, as those with an ammonia level of more than 40 μmol/L had a mean hospital stay of 17.6 days (SEM 3.39 days) vs. patients with normal levels who had a mean hospital stay of 9.62 days (SEM 1.1 days). This was statically significant, and the unpaired *t*-test result had a *p*-value of 0.0082.

**Figure 3:**
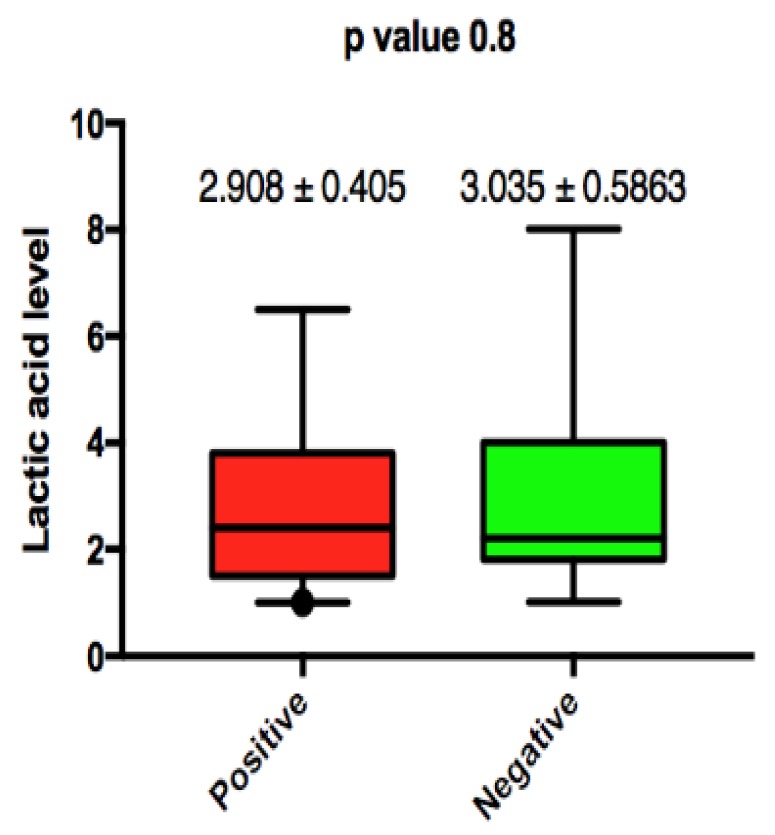
Association between lactic acid level on admission and microbial culture positivity.

**Figure 4:**
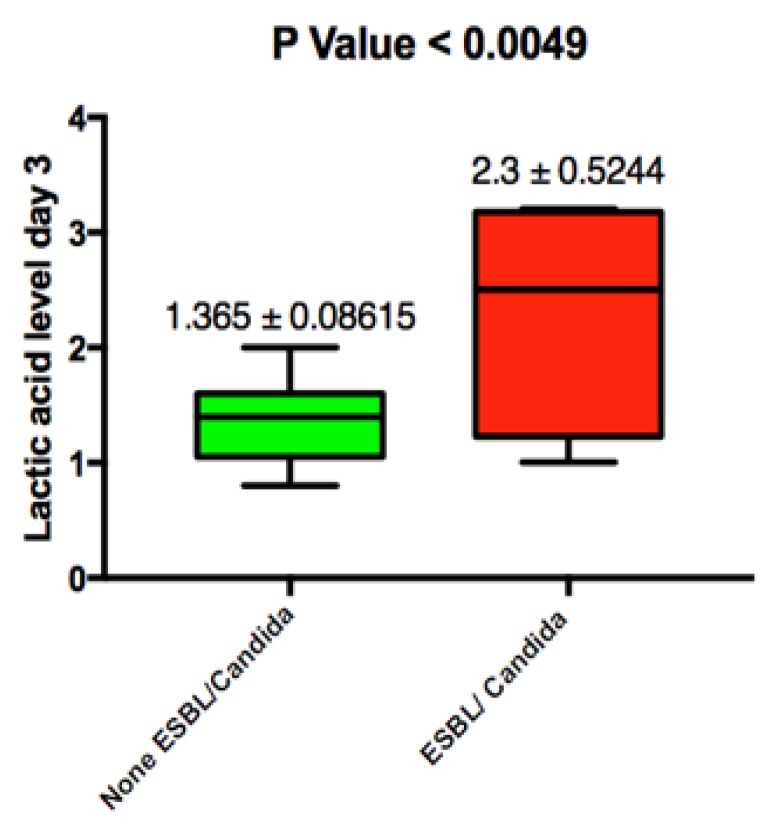
Lactic acid level on day 3 of study enrolment and its association with ESBL and candidial infection. ESBL: extended spectrum beta-lactamase producing organism. Values are presented as the following: mean ± standard error of mean (SEM). Statistically significant value is *p* < 0.05.

Lactic acid levels were persistently elevated in both patient groups on admission, and no significant difference was noted. Mean lactic acid levels in blood culture positive patients was 2.908 mmole/L ± 0.4 (SEM) vs. 3.035 mmole/L ± 0.5 (SEM); this was not statically significant with a *p* value of 0.8 (Figure 3). We did note that persistent elevation of lactic acid from admission until day 3 was associated with an ESBL/candidal infection (2.3 ± SEM 0.524 vs. 1.3 ± SEM 0.086 with *p*-value 0.0049) (Figure 4).

We evaluated the sensitivity and specificity of ammonia level 2-4 in association with a positive culture. Consistent with previous results, ammonia level 2-4 had a very high specificity of 90% (95% CI = 62–99%). This was not statistically significant and is attributed to the small sample size. The sensitivity of this test was low, with a value of 31%.

## 4. Discussion

We herein report the results of the ALPS study analyzing the association between elevated ammonia levels as a specific novel marker for predicting sepsis as per the SSG definition. Our results show a unique correlation between hyperammonemia measured within 36–48 h from admission and positive microbial culture. This was statistically significant and ammonia curves—despite having wide standard deviation bar for each value—were in concordance. This elevation coincides with the timing of positive microbial culture reporting from the laboratory. Thus, ammonia levels can be helpful in situations where antibiotics are started before the collection of blood cultures as we assume in these instances that they are more likely to be negative.

Lactic acid levels were elevated in both patient groups, and it had a poor ability in differentiating SIRS from sepsis. Our results showed a high specificity for elevated ammonia levels; thus, this test has the potential benefit of diagnosing patients with sepsis presenting with SIRS. On the other hand, ammonia level had a low sensitivity and was a poor test to rule out sepsis in patients with SIRS. We attribute this to the small sample size of patients with identified infection, i.e., the number of true positives was low. More studies are needed to explore the sensitivity of elevated ammonia levels in sepsis. Despite low sensitivity, elevated ammonia levels provided an advantage over lactic acid, which was elevated in both patient groups. This is particularly seen in shock, where hypoperfusion of tissues—rather than an infectious process—is the main reason for the production of shock.

During the design of the study, we aimed to find an association between ammonia levels and candida/ESBL infections. However, we were surprised that such infections were instead associated with elevated lactic acid levels within 36–48 h after admission and not on admission. This could be explained by the fact that such infections are not routinely covered by the empiric antibiotic regimen we had selected per our protocol. This means the infection would not have resolved and would have continue to cause an ongoing sepsis and lactic acid production. Given the low prevalence of such infections in our study, we question the utility of such results.

Hyperammonemia was associated with longer hospital stays, leading to an increase in morbidity and financial burden. However, a larger sample size is required to assess whether serum ammonia is a significant factor in predicting mortality in sepsis.

**Figure 5:**
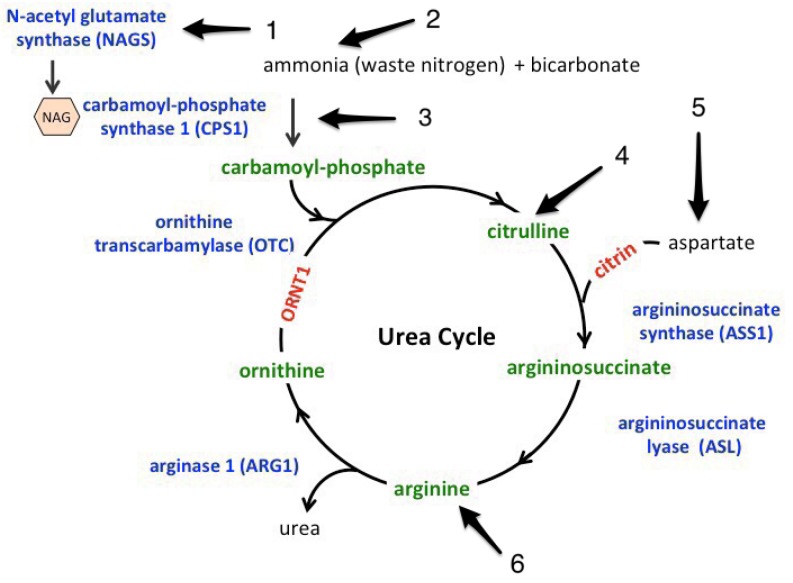
Effect of sepsis on urea cycle. (**1**) N-acetyl glutamate synthase is allosterically activated by arginine, which is depleted in sepsis secondary to decreased de novo synthesis. (**2**) Increased ammonia production secondary to increased proteolysis and defective transport into the mitochondria secondary to aquaporin-8 defect. (**3**) ATP is a cofactor of carbonyl phosphate synthase 1 (CPS1) and can lead to decreased CPS1 activity. (**4**) Decreased de novo synthesis of citrulline. (**5**) Decreased aspartate production secondary to decreased tricarboxylic acid (TCA) cycle activity in sepsis. (**6**) Decreased arginine in sepsis.

To the best of our knowledge, there have not been any randomized clinical trials done investigating the role of ammonia in sepsis. Sepsis affects intrinsic ammonia metabolism in several ways. These are summarized in Figure 5. The increase in lymphocytic glutaminase activity seen within 12 h from onset can lead to endotoxin-induced hyperammonemia [5]. Ammonia translocates into the inner mitochondrial membrane via aquaporin-8 mitochondrial channels, which are down-regulated in lipopolysaccharide-treated animal models, leading to impaired ureagenesis and causing hyperammonemia [6]. In addition, the rate-limiting step of the urea cycle mediated by carbonyl phosphate synthase 1 (CPS1) requires Adenosine triphosphate (ATP), which is depleted secondary to sepsis-induced cytopathic hypoxia [2]. The same step is also regulated by N-acetyl glutamate synthase (a co-enzyme) that is allosterically regulated by arginine, which is decreased in sepsis [7]. In addition, Gorge et al. concluded that Lipopolysaccharides (LPs)-induced inhibition of glutamate synthase located in the peri venous hepatic sinusoids that scavenge ammonia with maximal inhibition within 48 h of the onset of sepsis can lead to hyperammonemia in sepsis [8]. In addition to all the above mechanisms describing the effect of sepsis on the handling of ammonia, sepsis is considered a major catabolic state that leads to increased proteolysis [9]. This is associated with amino acid flux towards the liver [10], which increases the ammonia load [5]. The combination of decreased clearance and increase load are thought to be the major contributors of increased ammonia in sepsis.

Yet another mechanism is the increased extrinsic production of ammonia. Certain micro-organisms can increase production of ammonia in vivo secondary to increased urease splitting activity. Case reports have described noncirrhotic hyperammonemia in patients with sepsis secondary to Klebsiella [4].

Our study has several limitations. First, it is a single-institution study, and findings cannot be generalized given the unique population of patients treated in our hospital. Second, it is very difficult to completely exclude liver disease in septic patients. We depended on history provided by patients or family members and subclinical cirrhosis might have been present in some patients, especially those with hepatitis infection or who regularly consume alcohol.

The sample size is a known and obvious limitation of the study. This was due to the ethical concern of subjecting large number of patients to unproven novel test with no clinical data to support the rationale. Nonetheless, we managed to find an obvious correlation and the study can now be expanded to a multicenter trial to validate the data.

Lastly, the onset of sepsis was considered to be an emergency department admission. Time presentation after onset of sepsis was variable between patients and that could shift the peak ammonia level forward or backward and hence affect the results. Nonetheless, we believe our results do stand, given the statistical significance in the positive correlation between positive cultures and delayed ammonia level based on basic biochemical and metabolic data.

## 5. Conclusions

Extrapolated data from animal and human studies have led us to believe that sepsis can lead to hyperammonemia in the absence of an overt liver dysfunction. Metabolic derangements in protein, glutamine metabolism, ammonia transport, and ureagenesis are thought to be responsible. In addition, the timeline of these experiments has revealed that the nadir of these activities fell within 48 h of endotoxin induction, which coincides with the transient rise in ammonia level as shown in our study population. Hence, we conclude that ammonia has the potential of being utilized as a biomarker of sepsis-induced metabolic dysfunction. Larger and multicenter trials will be needed to verify our findings.

## Figures and Tables

**Table 1:** Basic characteristics for 30 patients divided by ammonia level of more than 40 μmol/L. All patients with presumed history of cirrhosis had a hepatitis C infection and had a normal aspartate aminotransferase (AST) and alanine aminotransferase (ALT) upon enrollment. No significant difference between groups could be determined due to the small sample size. APACHIE II (Acute Physiology and Chronic Health Evaluation II) is a score to estimate mortality of hospitalization—integer score from 0 to 71; a score of 20 carries a 40% mortality during hospitalization. * Cirrhosis is defined by the presence of clinical and/or laboratory stigmata of chronic liver failure such as but not limited to ascites or low albumin.

Variable	Abnormal Ammonia at Day 2-4 (*N* = 11)	Normal Ammonia at Day 2-4 (*N* = 19)
Age	58.3	58.5
Female Sex	45% (5)	31% (6)
Ammonia level on admission	35.7	32
White Blood Cell (WBC)	17.4	18.2
Procalcitonin	7.2	7.9
APACHIE II score	20	20
Average AST/ALT on admission	263/244	299/278
History of Liver cirrhosis *	27% (3)	15% (3)
Creatinine	1.85	1.8
Vasopressor requirement	100% (8)	50% (11)
Mechanical ventilator	25% (2)	18% (4)
Death	18% (2)	36.8 (7)

**Table 2:** Source of infection in patients enrolled in the study.

Source of Infection	Percentage % (*N*)
Pneumonia	50% (15)
Urinary tract infection	13.3 (4)
Infective endocarditis	6.66% (2)
Susceptive sepsis with no source	30% (9)
